# Magnesium sulphate for fetal neuroprotection: benefits and challenges of a systematic knowledge translation project in Canada

**DOI:** 10.1186/s12884-015-0785-8

**Published:** 2015-12-22

**Authors:** Katherine C. Teela, Dane A. De Silva, Katie Chapman, Anne R. Synnes, Diane Sawchuck, Melanie Basso, Robert M. Liston, Peter von Dadelszen, Laura A. Magee, for the MAG-CP Collaborative Group

**Affiliations:** Seattle University College of Nursing, Seattle, Washington USA; Department of Obstetrics & Gynaecology, University of British Columbia, Vancouver, Canada; Child and Family Research Institute, University of British Columbia, Vancouver, Canada; Department of Medicine, University of British Columbia and British Columbia Women’s Hospital and Health Centre, Vancouver, Canada; Division of Neonatology, Department of Paediatrics, University of British Columbia, Vancouver, Canada; University of London, Cranmer Terrace, Rm J0.27, Jenner Wing, St. George′s, SW17 London, 0RE UK

**Keywords:** Knowledge translation, Magnesium sulphate, Fetal neuroprotection, Preterm birth

## Abstract

**Background:**

Administration of magnesium sulphate (MgSO_4_) to women with imminent preterm birth at <34 weeks is an evidence-based antenatal neuroprotective strategy to prevent cerebral palsy. Although a Society of Obstetricians and Gynaecologists of Canada (SOGC) national guideline with practice recommendations based on relevant clinical evidence exists, ongoing controversies about aspects of this treatment remain. Given this, we anticipated managed knowledge translation (KT) would be needed to facilitate uptake of the guidelines into practice. As part of the Canadian Institutes of Health Research (CIHR)-funded MAG-CP (MAGnesium sulphate to prevent Cerebral Palsy) project, we aimed to compare three KT methods designed to impact both individual health care providers and the organizational systems in which they work.

**Methods:**

The KT methods undertaken were an interactive online e-learning module available to all SOGC members, and at MAG-CP participating sites, on-site educational rounds and focus group discussions, and circulation of an anonymous ‘Barriers and Facilitators’ survey for the systematic identification of facilitators and barriers for uptake of practice change. We compared these strategies according to: (i) breadth of respondents reached; (ii) rates and richness of identified barriers, facilitators, and knowledge needed; and (iii) cost.

**Results:**

No individual KT method was superior to the others by all criteria, and in combination, they provided richer information than any individual method. The e-learning module reached the most diverse audience of health care providers, the site visits provided opportunity for iterative dialogue, and the survey was the least expensive. Although the site visits provided the most detailed information around individual and organizational barriers, the ‘Barriers and Facilitators’ survey provided more detail regarding social-level barriers. The facilitators identified varied by KT method. The type of knowledge needed was further defined by the e-learning module and surveys.

**Conclusions:**

Our findings suggest that a multifaceted approach to KT is optimal for translating national obstetric guidelines into clinical practice. As audit and feedback are essential parts of the process by which evidence to practice gaps are closed, MAG-CP is continuing the iterative KT process described in this paper concurrent with tracking of MgSO_4_ use for fetal neuroprotection and maternal and child outcomes until September 2015; results are anticipated in 2016.

**Electronic supplementary material:**

The online version of this article (doi:10.1186/s12884-015-0785-8) contains supplementary material, which is available to authorized users.

## Background

Preterm birth is the leading cause of infant death, illness, and disability in Canada and worldwide. Despite marked improvements in survival rates of preterm infants, the risk of neurodevelopmental impairment, including cerebral palsy (CP), is substantial (2–2.5/1000 live births) and has not decreased over recent years [1, 2].

Magnesium sulphate (MgSO_4_) for fetal neuroprotection provides one of the first prenatal evidence-based treatments to improve the neurodevelopmental outcomes of children born at <34 weeks, [3–5] and international guidelines were quick to recommend this therapy [6, 7]. However, unresolved controversies were recognized [6]. First, high MgSO_4_ doses formerly used for tocolysis were associated with neonatal hypermagnesemia, hypotonia, resuscitation, and mortality [8], but no such effects were seen when MgSO_4_ was used at lower doses for fetal neuroprotection in the relevant trials or in subsequent publications [9]. Second, the mechanism of MgSO_4_ action was not understood. Third, the gestational age threshold for optimal MgSO_4_ benefit in the neuroprotective trials was not clear below <34 weeks. Finally, there were inadequate studies of long-term outcomes, although subsequent publications have been reassuring [10, 11].

It is recognized that knowledge translation (KT) and mobilization is an iterative process, and that dissemination of information is usually not enough to change practice or behaviour. In obstetrics, a notable example of failing to bridge the gap between knowledge and practice was the 22-year lag between the publication on antenatal corticosteroid use and the National Institutes of Health consensus conference and American College of Obstetricians and Gynecologists (ACOG) Committee Opinion on the topic [12, 13].

We anticipated that effective uptake of MgSO_4_ use for fetal neuroprotection would require an effective process of ‘managed knowledge translation (KT)’, whereby evidence is reviewed to avoid misinformation, individual and institutional barriers to implementation are identified and overcome, and ‘real-world’ effectiveness and safety are monitored [14–17]. Consistent with its mandate [18] CIHR funded the MAG-CP managed KT project to actively implement the relevant Canadian guidelines into clinical practice [6], as done for pregnancy hypertension guidelines in British Columbia [19].

MAG-CP is using a combination of active and passive KT methods to educate health care providers, and to better understand their knowledge, beliefs and practices related to the use of MgSO_4_ for fetal neuroprotection and their organizational culture. This paper focuses on three primary KT methods designed to impact both individual health care providers and the organizational systems in which they work: (i) an interactive online e-learning module with opportunity to identify potential barriers to uptake; (ii) site visits to participating tertiary perinatal units of the Canadian Perinatal Network (CPN) for discussion of the evidence and potential facilitators and barriers; and (iii) circulation of an anonymous survey at CPN sites for the systematic identification of facilitators and barriers for uptake of practice change. We sought to compare these three strategies, highlighting the relative advantages and disadvantages of each approach, in order to offer recommendations on timely and cost-effective KT implementation [15].

## Methods

MAG-CP is a managed KT project approved in 18 of Canada’s 23 tertiary perinatal units that are part of the CPN. Central approval for MAG-CP was obtained from both the University of British Columbia’s (H11-02214) and at each site’s Research Ethics Board (see Additional file 1: Table S2), for both KT activities and audit of MgSO_4_ utilization and health outcomes through the CPN and the Canadian Neonatal Follow-up Network (CNFUN). Written consent was not required for conducting educational activities as part of the educational component of the project. All health care providers who participated did so voluntarily as with any other educational activities in the hospital, and they voluntarily and anonymously participated in the e-learning module and the barriers & facilitators questionnaire.

MAG-CP aims to determine, for pregnancies at risk of imminent preterm birth at <29 weeks, whether utilization of MgSO4 for fetal neuroprotection: (i) can be increased over 4 years to a pre-specified level of 80 %; (ii) is associated with the anticipated decrease in CP and ‘death or CP’ that was seen in RCTs; and (iii) is associated with an acceptable safety profile for both mothers and babies/children. The project focuses on babies at risk of being born at <29 weeks because (i) they are at highest risk of CP; and (ii) they are those for whom maternal interventions and maternal and perinatal outcomes are collected directly by the CPN or through linkage to the CNFUN that tracks neurodevelopmental outcomes to three years of age. Outcome data collection is ongoing.

In our initial year of activity, MAG-CP used three primary methods of KT: an interactive, online ‘e-learning module’, visits to CPN sites participating in the MAG-CP project [20], and a ‘Barriers and Facilitators (B&F) Survey’ that was distributed and collected by each site’s local team. This knowledge translation project, including the design and timing of site visits and B&F questions, was informed by the concepts and principles of Roger’s Innovation-Diffusion theory. These include elements of: the complexity of the innovation, characteristics of adopters, communication channels, time considerations for adoption and uptake, and organizational characteristics of the social system. At the individual level, the process of behaviour change includes knowledge of the innovation, persuasion for uptake, an individual decision for uptake and use at which point the innovation is either accepted or rejected, implementation of the innovation, and finally, confirmation in their decision for uptake [21]. Our qualitative data collection and analyses were designed to reflect the consolidated criteria for reporting qualitative research (COREQ) checklist [22].

- *E-learning module*

A commercially-managed e-learning module (AdvancingIn®) was created by the Central MAG-CP Team and AdvancingIn® [23] to reach all health care professionals in a widely dispersed Canadian clinical community. This approach focused on cognitive learning and problem solving for the individual related to MgSO_4_ for neuroprotection, and principles in adult education that have been effective in enhancing knowledge and confidence in practice change [24–27].

The Module contained basic questions about the participants’ characteristics, including hospital site (location and size) and type of care provider/role in that hospital. The content of the Module consisted of: (i) pre-test questions and a survey (to stimulate recognition of knowledge gaps and opinions), (ii) a concise summary of relevant background and published evidence for the effectiveness of MgSO_4_ for fetal neuroprotection (including the relevant SOGC Clinical Practice Guideline [6], a summary of controversies and uncertainties, and other relevant reading materials including each of the three relevant meta-analyses [3–5]), (iii) case analyses (to test application of knowledge and simulate situations found in clinical practice), (iv) practice tools, such as pre-printed orders for MgSO4 administration for fetal neuroprotection, a pocket card to distinguish ‘imminent’ from ‘threatened’ preterm birth, and an information sheet about how to counsel a patient about the potential benefits of MgSO_4_ as a neuroprotective agent (as many are unfamiliar about the nature of the outcome of CP), (v) post-test questions and a survey (designed to test knowledge, identify any remaining gaps, and explore opinions about knowledge and practice), (vi) a course evaluation, and (vii) a discussion forum in which participants provided comments and views about MgSO4 use. Details of participant characteristics (as above), and free-text response questions from the discussion forum and course evaluation (Additional file 2: Panel S1) were used for analysis. (The effectiveness of the module as a learning tool will be reported separately.)

The link for the MAG-CP e-learning Module was actively disseminated by email to SOGC members from Mar 11/2012 to Mar 10/2013 by AdvancingIn®, and to MAG-CP sites by the Central MAG-CP Team. The Module was accessible free of charge, and continuing medical education points were offered for completion. (On Mar 11/2013, the Module was migrated over to the CPN website for ongoing use by those at MAG-CP sites [28]).

Costs accounted for were related to the AdvancingIn® invoice and did not include Central MAG-CP Team time.

- *Site Visits at participating CPN sites*

Small group discussions were planned by the Central MAG-CP Team for the local team at each participating site (Mar to Dec/12). This approach was designed to both reinforce cognitive learning, and facilitate interaction, discussion, and problem solving at inter-disciplinary and organizational levels, in order to address beliefs, practices, and culture.

These on-site facilitated sessions were planned to occur shortly after local approvals for the project were in place. One Central Team member attended all visits (LAM), always accompanied by at least one other Team member (AS, DS, or MB). Local site members were asked to complete the e-learning module prior to the site visit, and to encourage their local health care practitioners to do the same.

Sessions were planned to be 3–4 hours in duration involving didactic and interactive components, with a flexible agenda so that the local team could prioritize their needs. Planned didactic material was comprised of a presentation (ideally at grand rounds in obstetrics) of the evidence about MgSO_4_ for fetal neuroprotection, the national guideline and summary of recommendations (2011) [6], and a synopsis of CPN and CNFUN methods that would enable audit of the use of MgSO_4_ for fetal neuroprotection and associated relevant outcomes. The planned interactive component consisted of a small-group discussion between the Central MAG-CP and local teams to review points raised at the rounds or by the local B&F Survey (see below), and identify potential local barriers and facilitators to practice change.

Attendance, professional designation, and minutes of the discussion were collected at each visit. A full report was written within one week of the visit by the facilitator in attendance at all visits (LAM). The Central and local team members in attendance were given the opportunity to revise the report which when final, was circulated to the local site team.

Costs accounted for related to transportation, accommodation, and printing of presentations and practice tools necessary for distribution to the attendees. Time by members of the Central MAG-CP Team was not included, as this was covered by academic research time as University of British Columbia (UBC) employees.

- *Barriers and Facilitators (B&F) Survey*

An anonymous B&F survey was designed to explore perceived barriers and facilitators of implementation of MgSO_4_ for fetal neuroprotection (Additional file 1: Table S3). This approach was chosen to determine overall organization readiness and address potential challenges, as well as identify knowledge gaps and opinions. Site teams were encouraged to review their own reports prepared by the Central MAG-CP Team to stimulate reflection and action.

Individual, institutional, and social aspects of KT were considered. The B&F survey was available in English or French, in paper or electronic PDF format. The B&F surveys were distributed to individuals involved in the care of women eligible for MgSO_4_ for fetal neuroprotection and their babies. Responses were collected by each site team. Sites were encouraged to make every attempt to complete the questionnaire prior to, or within three months following, the site visit, and then every six months thereafter. Data presented here are from each sites’ baseline survey.

Costs were considered to be negligible, related to printing of the two-page surveys if an electronic PDF was not used to capture responses. As local team members were hospital employees, their time was not included in costing.

- *Analyses*

NVivo qualitative research software (QSR International Pty Ltd. Version 10, 2012) was used to organize, analyze, and interpret data from the three KT methods. Data, which included descriptive information about respondents, were coded and stored as nodes within NVivo, which were subsequently used to explore similar and disparate themes that emerged between the formats.

Qualitative research is an iterative and malleable process, with coding and nodes consistently refined throughout the analysis. Due to the possibility of introducing bias by a member of the central MAG-CP team, two independent reviewers (KC and then KCT) conducted the analyses. To further validate the analyses, a blind-coding of the Barriers & Facilitators Survey was conducted by a member of the central MAG-CP team (DAD) to identify discrepancies. The results of this activity showed that themes were consistent (Additional file 1: Table S4), and thereafter, the remainder of the analyses were carried out by the independent reviewers.

Findings were divided into three main categories: barriers to implementation, facilitators to implementation, and knowledge needed (for respondents to more comfortably and easily implement the intervention). To better interpret themes and implications, barriers and facilitators were divided into three basic categories: individual-level, institutional-level, and social-level. The final list of coding nodes is shown in Additional file 1: Panel S2 and Additional file 1: Panel S3. Graphical and tabular representations of the findings were created from the emerging patterns and themes according to province or health care provider, as well as size of the tertiary centre.

## Results

In the first year of MAG-CP activity (March 2012–3), 14/18 sites contributed to this assessment of initial KT activity for the site visits and B&F survey: Women’s Health Program (Newfoundland), IWK Health Centre (Nova Scotia), The Moncton Hospital, Saint John Regional Hospital, and Dr. Everett Chalmers Hospital (New Brunswick), Centre Hospitalier de l’Universite Laval (Quebec), Mount Sinai Hospital, Sunnybrook Health Sciences Centre, Saint Joseph’s Health Centre, and The Ottawa Hospital (Ontario), Royal University Hospital (Saskatchewan), Foothills Medical Center, and the Royal Alexandra Hospital (Alberta), and British Columbia Women’s Hospital and Health Centre (British Columbia). Two additional centres had participants complete the e-learning module only: Regina General Hospital (Saskatchewan) and Victoria General Hospital (British Columbia).

- *Methods of KT utilized*

In the first year of MAG-CP, 8/18 sites utilized all three methods of KT, 6/18 utilized two methods (3 e-learning module and site visit, 1 e-learning module and B&F survey, and 2 site visit and B&F survey), 2/18 sites used only the e-learning module, and 2/18 used none of the KT methods offered.

The e-learning module was completed by staff at 14/18 MAG-CP sites, with representation from all major regions in Canada except for the Northwest Territories and Nunavut.

The 10 site visits covered 13/18 (72.2 %) MAG-CP sites of the major provinces; one site declined a site visit and it could not be arranged at a mutually convenient time at 4 others. All but one site visit involved didactic rounds and all involved an interactive session. The median attendance was 9, and ranged from 5–50.

Twelve of the eighteen sites completed the B&F survey, 5 (41.7 %) before the site visit. There was a median [IQR] of 17 [15, 19] surveys completed for small, 14.5 [11, 18] for medium, and 14.5 [13, 21] for large CPN sites.

- *Characteristics of participants (Table*1*)*

**Table 1:** Characteristics of 1608 participants by type of KT method (N (%) or mean ± SD of column total unless otherwise specified)

	e-learning module (*N* = 1274)	Site visits (*N* = 146*)	Barriers & Facilitators Survey (*N* = 188†)
Health care provider type			
Physicians	458 (35.9 %)	59 (40.4 %)	92 (48.9 %)
* Maternal-Fetal Medicine*	- ‡	18	33
* General Obs/Gyn*	168	26	37
* General Practice*	200	0	0
* Anaesthesia*	15	4	2
* Pediatrics/Neonatology*	75	11	11
* Unclassified*	0	0	9
Nurses	439 (34.5 %)	57 (39.0 %)	87 (46.3 %)
* Registered nurse*	412	56	80
* Nurse practitioner*	25	1	0
* Licensed practical nurse*	3	0	0
* Unclassified*	0	0	7
Other	177 (13.9 %)	20 (13.7 %)	7 (3.7 %)
* Midwife*	4	1	2
* Medical resident*	9	3	2
* Medical student*	7	1	0
* Research staff*	5	14	1
* Pharmacist*	150	1	1
* Administration*	2	0	1
Unclassified	200 (15.7 %)	10 (6.8 %)	2 (1.1 %)
Province/Territory			
British Columbia	162 (12.7 %)	10 (6.8 %)	21 (11.2 %)
Alberta	234 (18.4 %)	80 (54.8 %)	15 (8.0 %)
Saskatchewan	45 (3.5 %)	8 (5.5 %)	16 (8.5 %)
Manitoba	50 (3.9 %)	0	0
Ontario	517 (40.6 %)	25 (17.1 %)	58 (30.9 %)
Quebec	87 (6.8 %)	5 (3.4 %)	14 (7.4 %)
Newfoundland	35 (2.7 %)	6 (4.1 %)	17 (9.0 %)
New Brunswick	82 (6.4 %)	12 (8.2 %)¶	34 (18.1 %)
Nova Scotia	35 (2.7 %)		13 (6.9 %)
Prince Edward Island	6 (0.5 %)	0	0
Yukon	6 (0.5 %)	0	0
NWT	9 (0.7)	0	0
Nunavut	5 (0.4)	0	0
Unknown	1 (0.0 %)	0	0
Works in a tertiary perinatal unit	472 (37.0 %)	146 (100 %)	188 (100 %)
Small (<3000 del/yr)	128/472 (27.1 %)	13/146 (8.9 %)	51/188 (27.1 %)
Medium (3000–4999 del/yr)	52/472 (11.0 %)	48/146 (32.9 %)	29/188 (15.4 %)
Large (≥5000 del/yr)	291/472 (61.7 %)	85/146 (58.2 %)	108/188 (57.5 %)
Missing	1/472 (0.2 %)	0	0

NWT (Northwest Territories), Del (deliveries), KT (knowledge translation), Obs/Gyn (Obstetrics and Gynaecology)

Note that the total % is broken down in various ways; each area until a bolded row adds to 100 %

* 13 sites were covered by 10 sites visits, as three small sites joined the visit at a larger centre

† Questionnaires were completed at 12/18 MAG-CP sites

‡ Not available as an option in the e-learning module

¶ Representatives from New Brunswick and Nova Scotia attended the site visit in Nova Scotia

There were 1608 responses in the three forms of KT: 1274 (79.2 %) respondents to the e-learning module (Mar 2012–3), 146 (9.1 %) participants at 10 sites visits (Mar-Dec/12), and 188 (11.7 %) respondents to the B&F Survey from 12 sites (Jun/12-Mar/13).

Table 1 shows that physicians comprised the highest percentage of respondents across each KT method, followed by nurses and then by “other”; 200 (15.7 %) of e-learning module respondents did not identify with a health care provider type listed. As all project related staff were included in the site visits, more research staff participated in that format compared with the e-learning module and B&F survey, which were largely distributed to clinicians. The greatest number of pharmacist participants was seen in the e-learning module as this format was accessible to all staff whereas the site visits and B&F survey were more limited to clinicians ordering and implementing the protocol, or directly involved in the MAG-CP study. The greatest proportion of respondents for the e-learning module and B&F survey were from Ontario, and for the site visits, Alberta. Only the e-learning module accessed individuals outside of tertiary perinatal units. Small MAG-CP sites were underrepresented at site visits, and for each KT method, most respondents were from large perinatal centres.

- *Costs*

The cost for developing the e-learning module was $10,000.00, which was a reduced rate provided for working collaboratively with SOGC. The total cost of transportation and accommodations to conduct the site visits was $17,417.08, approximately $1,750.00 per visit. Costs related to the B&F survey were assumed to be negligible (see Methods).

- *Barriers identified (Table*2*)*

**Table 2:** Barriers to use of MgSO4 for fetal neuroprotection (N (%) responses for all responses that relate to barriers)

Nodes	Sub-nodes	e-learning module (*N* = 119)	Site visits (*N* = 92)	Barriers & Facilitators Survey (*N* = 147)
Individual-level		51 (42.9 %)	47 (51.1 %)	86 (58.5 %)
Unsupportive attitudes and beliefs		12 (10.1 %)	3 (3.3 %)	12 (8.2 %)
Not within provider’s control		4 (3.4 %)	0	2 (1.4 %)
No experience		3 (2.5 %)	1 (1.1 %)	3 (2.0 %)
Inadequate knowledge and understanding	Overall	15 (12.6 %)	18 (19.6 %)	35 (23.8 %)
	* In self*	0	3	18
	* In others*	13	4	9
	* Unclear who lacks knowledge*	2	11	8
Forgetting to administer MgSO4		1 (0.8 %)	1 (1.1 %)	3 (2.0 %)
Fears	Overall	14 (11.8 %)	16 (17.4 %)	13 (8.8 %)
	* Legal*	0	2	3
	* Medication error*	0	2	0
	* Adverse effects of withholding MgSO*_*4*_	0	3	5
	* Adverse effects of MgSO*_*4*_	14	9	5
Failure to implement guidelines		0	1 (1.1 %))	4 (2.7 %)
Evidence concerns (sufficiency and validity)		2 (1.7 %)	7 (7.6 %)	14 (9.5 %)
Institutional-level		64 (53.8 %)	34 (37.0 %)	40 (27.2 %)
Unsupportive institutional culture		2 (1.7 %)	3 (3.3 %)	3 (2.0 %)
Timing and transport		20 (16.8 %)	17 (18.5 %)	6 (4.1 %)
Resource constraints		31 (25.2 %)	12 (13.0 %)	20 (13.6 %)
Policy development and implementation		11 (9.2 %)	2 (12.0 %)	11 (7.5 %)
Social-level		4 (3.4 %)	11 (12.0 %)	21 (14.3 %)
Lack of provider-institutional consensus		1 (0.8 %)	4 (4.3 %)	15 (10.2 %)
Inadequate interprovider communication		0	7 (7.6 %)	5 (3.4 %)
Educating patients		3 (2.5 %)	0	1 (0.7 %)

MgSO_4_ (magnesium sulphate)

Note that the total % is broken down in various ways; each area until a bolded row adds to the % listed in the bolded row

By all KT methods, individual-level barriers were very common, particularly inadequate knowledge and understanding, and fears. Some participants described multiple barriers while others did not mention any, leading to a discordance in response totals for Table 2 compared with overall participation listed per method in Table 1.

Inadequate knowledge was identified in the e-learning module as relating to others most often (“*Not all staff are informed of the standards”* – RN, Ontario), versus to self most often in the B&F survey (18/35, 51.4 % relevant responses, 13 of which came from individuals who completed the B&F survey prior to the site visit. “*I need a course on it or guideline*” – Physician, New Brunswick). At site visits, the report did not contain sufficient detail about who was lacking knowledge (self versus others). Evidence concerns with regards to sufficiency and validity were most evident in response to the B&F survey (“*Evidence is suggestive, but not overwhelming in terms of using*” – Neonatologist, Nova Scotia), with 7/14 (50 %) of similar responses from individuals who completed the survey prior to the site visit. 10/14 responses regarding evidence concerns were from RNs, with OB/GYNs (2/14) and neonatologists (2/14) represented less. Both neonatologists and two nurses who reported evidence concerns suggested a lack of validity, whereas the rest of the responses (10/14) were concerning the sufficiency of the evidence. Similar concerns were documented at site visits (“…*variable belief in the evidence, with some strong local opinion leaders who philosophically questioned the validity of meta-analysis in informing clinical practice guidelines…*” – site visit report, Alberta) and the e-learning module *(”I am concerned that MgSO4 is not as benign as is described in the literature or this document”*). Fears were commonly reported across all KT methods, with the fear of adverse effects of MgSO_4_ most often reported. The site visits offered the broadest variety of distinct fears, being the only format to include ‘fear of medication error’ specifically. The e-learning module offered the least variety of fears, with only ‘fear of adverse effects of MgSO_4_’ identified. Stated unsupported attitudes referred nearly always to others (“*Some ObGyn are close-minded*” – Nurse, Quebec), and the richness of responses in this sub-node in the e-learning module and B&F survey enabled identification of similar concerns between professional groups at 3/10 (30 %) site visits at Western and Eastern Canadian locations.

Institutional-level barriers were also commonly expressed, though less so in the B&F survey relative to the other formats. Timing and transport issues around MgSO_4_ administration, as well as resource constraints on busy delivery suites were detailed most commonly, followed by the need for policy development and implementation.

Social-level barriers were uncommon throughout, but most were reported in the B&F survey, particularly a lack of provider-institutional consensus.

- *Facilitators (Table *3*)*

**Table 3:** Facilitators of use of MgSO4 for fetal neuroprotection (N (%) responses for all responses that relate to facilitators)*

Nodes	Sub-nodes	Site visits (*N* = 128)	Barriers & Facilitators Survey (*N* = 171)
Individual-level		17 (13.3 %)	38 (22.2 %)
Supportive attitudes and beliefs		4 (3.1 %)	9 (5.3 %)
Knowledge and understanding		8 (6.3 %)	15 (8.8 %)
Early adopters/mobilizers		1 (0.8 %)	9 (5.3 %)
Comfort/experience using MgSO4		4 (3.1 %)	5 (2.9 %)
Institutional-level		80 (62.5 %)	107 (62.6 %)
Policies and protocols	Overall	34 (26.6 %)	26 (15.2 %)
	* Pre-printed orders for MgSO4 use*	5	6
	* Pre-mixed bags of MgSO4*	8	2
	* Mechanism for audit and feedback*	8	3
	* Unclassifiable*	13	15
Local champion/opinion leader		8 (6.3 %)	11 (6.4 %)
Facility characteristics	Overall	38 (29.7 %)	70 (40.9 %)
	* Supportive institutional culture/evidence-based*	18	38
	* Patient load*	3	10
	* Human resource capacity*	5	4
	* Education and professional development*	12	18
Social-level		31 (24.2 %)	26 (15.2 %)
Patient voice/awareness		4 (3.1 %)	2 (1.2 %)
Knowledge translation		17 (13.3 %)	5 (2.9 %)
Community support		2 (1.6 %)	0
Communication and collaboration		8 (6.3 %)	19 (11.1 %)

* When the e-learning module was designed, a specific exploration of facilitators was not planned and therefore, undertaken

Response about general policy/protocol and not specific to pre-printed orders, pre-printed bags, or audit/feedback

Note that the total % is broken down in various ways; each area until a bolded row adds to the % listed in the bolded row

Facilitators of guideline adoption were included specifically for discussion at the site visits and the B&F survey, but not in the e-learning module which was developed first. Some participants described multiple facilitators while others did not mention any, leading to a discordance in response totals for Table 3 compared with overall participation listed per method in Table 1.

Institutional-level facilitators that were most commonly reported included: facility characteristics, particularly a supportive institutional culture and evidence-based practice and education and professional development, followed by policies and protocols. Experience and comfort with use of MgSO_4_ for use in pre-eclampsia/eclampsia was more frequently reported as a facilitator than a barrier, whereas *inadequate* knowledge regarding neuroprotection was more often a barrier. Few respondents identified a local champion/opinion leader as important. Individual- and social-level facilitators were less commonly identified. For both formats, patient-voice was seen as a facilitator rather than a barrier to administration of MgSO_4_ for neuroprotection, despite fears of negative side effects on the patient being listed as a barrier.

- *Nature of the knowledge needed (Table*4*)*

**Table 4:** Responses for ‘knowledge needed’ (N (%) responses) for all responses that relate to knowledge needed)

Nodes	Sub-nodes	Sub-sub-node	e-learning module (*N* = 188)	Site visits (*N* = 85)	Barriers & Facilitators Survey (*N* = 65)
Mechanism of action			11 (5.9 %)	2 (2.4 %)	17 (26.2 %)
Administration	Overall		47 (25.0 %)	61 (71.8 %)	26 (40.0 %)
	Transfer		0	2	0
	Threatened preterm labour vs. imminent preterm birth		3	10	0
	Timing of administration		6	9	7
	Standards of practice		4	10	2
	Re-treatment		0	7	3
	Pre-printed orders		1	0	0
	Policies and protocols		8	3	7
	Multiple pregnancies		1	0	0
	Gestational age		8	7	2
	Drug interactions		5	0	0
	Contraindications		1	2	0
	Unclassified		10	11	5
Side effects and risks	Overall		26 (13.8 %)	16 (18.8 %)	6 (9.2 %)
	Rapid delivery		1	0	0
	Overuse		1	1	0
	Interventions as a result of MgSO4		3	0	0
	Increased monitoring needed		5	0	0
	Adverse physiological effects – neonate	* Toxicity*	3	1	0
		* Problems with feeding*	1	0	0
		* Neonatal respiratory depression*	6	3	0
		* Long-term effects*	0	1	2
		* Adverse neurological effects*	1	0	0
	Adverse physiological effects – general (unspecified maternal/neonate)		0	7	0
	Unclassified		4	2	4
KT tools	Overall		7 (3.7 %)	0	3 (4.6 %)
	Audit and feedback		1	0	0
	Unclassified		6	0	3
Research	Overall		46 (24.5 %)	5 (5.9 %)	13 (20.0 %)
	Further research		33	3	5
	Evidence to date		6	1	7
	Unclassifiable		7	1	1
Other uses and topics (not MgSO_4_ for fetal neuroprotection)			30 (16.0 %)	1 (1.2 %)	0
None stated			21 (11.2 %)	0	0

MgSO_4_ (magnesium sulphate)

Note that the total is broken down in various ways; each area until a bolded row adds to the total n listed in the bolded row

As inadequate knowledge was identified as a barrier to implementation, all three KT formats contained rich information on the *nature* of the knowledge gaps. We report these separately as overall lessons.

Issues around how to administer MgSO_4_ for fetal neuroprotection came up most frequently in all KT methods, particularly so in site visit discussions. Most commonly, respondents identified the following aspects of administration as requiring clarification: eligibility (i.e., diagnosing threatened vs. imminent preterm birth), timing of administration relative to delivery, standards of practice, policies and protocols, and the gestational age for eligibility. The variety of responses was richest for the e-learning module (9 sub-nodes) and site visits (8 sub-nodes) compared with the B&F survey (5 sub-nodes).

Side effects and risks were commonly identified as a learning need by respondents, particularly in the e-learning module (5 sub-nodes and 4 sub-sub-nodes) and site visits (3 sub-nodes and 3 sub-sub-nodes) compared with the B&F survey (1 sub-node and 1 sub-sub-node). Knowledge needed regarding adverse effects focused primarily on the neonate. The following comments illustrate the variety of detail observed.

*“When neonatal respiratory depression occurs as a suspected result of maternal magnesium sulfate administration, is calcium gluconate recommended to assist in resuscitation, or would that counteract the effects?” – RN, e-learning module*

*“The relationship between maternal dosing and the potential impact on fetal heart rate variability. Specifically, would 2 g/hr have an impact on FHR variability.” – Site visit report, Saskatchewan*

*“Follow-up of patient care – mother and neonate – statistics if possible” – Physician, B&F survey*

While many respondents specified that further knowledge and/or research is needed, more than 10 % of responses from the e-learning module and B&F survey identified “further research” as being needed without identifying a research question.

- *Comparison of the three KT methods (Table*5*)*

**Table 5:** Summary of key results / findings unique to each KT format

	e-learning module	Site visits	B&F survey
Respondents accessed	• Reached the largest number of participants across the widest geographic area	• Reached fewer practitioners, but of a similar scope in terms of roles within health care	• Most limited in terms of ‘other’ non-physician and non-nurse respondents
Barriers	• Most restricted breadth of fears listed • Insufficient knowledge most often identified in others	• Greatest spread of barriers across individual, institutional, and social levels	• More social-level barriers compared with other formats • Insufficient knowledge most often identified in respondent
Facilitators	-*	• Institutional-level most cited, followed by *social*-level	• Institutional-level most cited, followed by *individual*-level
Knowledge needed	• Greatest number of responses calling for further research	• Information on administration cited most often	• Least amount of information provided
Method	• One-way	• Two-way	• One-way
Approximate cost	• $10,000	• $17,500 (total) or $1750 per visit†	• Negligible (assumption)†

B&F (Barriers and Facilitators)

* Facilitators were not included in the e-learning module format which was developed first

† As academic health care centre employees, salaries were considered to cover educational activities such as for MAG-CP. Site visit costs were based on travel expenses

No one KT method was superior to the others by all criteria. The e-learning module reached the widest audience of health care providers, the site visits provided opportunity for iterative dialogue, and the B&F survey was the cheapest. However, although the variety of barriers identified was greatest for the site visits, the B&F survey provided more information around social-level barriers. The type of knowledge needed was further defined by the e-learning module and B&F surveys. Also, facilitator type differed by KT method.

## Discussion

### Our findings

Of the three KT approaches taken in our managed KT project MAG-CP, not surprisingly, the online e-learning module reached a greater number of individuals, but all three KT methods accessed views from similar types of care providers. In part, this may have been a result of the Central MAG-CP directive that site visit members and respondents to the B&F survey should be a representative population.

No individual KT method was superior to the others in terms of the barriers and facilitators identified, and knowledge needed determined by rates and richness of qualitative data. Although we did not see a drop in length/quality of quotes with the rise in breadth/variety, the site visits provided the most detailed information about individual and organizational barriers, and the ‘Barriers and Facilitators’ on-site survey more detail about social level barriers. The facilitators identified varied by KT method. The type of background, evidential, clinical, and judgment knowledge needed was further defined by the e-learning module and surveys.

It is important to recognize the advantages and restrictions associated with each KT strategy. The e-learning module provided intellectual stimulation that the learner could move through at his/her own pace, in the form of online education documents, case studies, questions and answers, and tools, all presented prior to the discussion forum which sought learners’ feedback; this may have prompted the wide variety of information obtained, suggesting that online KT is effective. Although the site visits started with a presentation of the evidence, it was limited by time and event structure (usually a didactic round). However, issues raised in the site visit report may have been a summary of a larger conversation, stimulated by the facilitators having the opportunity to ask probing questions to clarify views and enrich the discussion. Also, there was no limit on the range of topics discussed in contrast with the e-learning module and B&F survey, which had pre-set questions, such as the e-learning module with a more restricted interest in ‘fears’. On the other hand, statements from the e-learning module and B&F survey are direct quotes in response to open text boxes, without imposing limitations on time or space (although other limitations may have been at play, such as personal schedules or end-of-survey fatigue). These formats have the additional potential advantage of anonymity, possibly a reason for fewer comments about “unsupportive attitudes and beliefs” in site visit reports and more about social barriers on the B&F survey. This would support having an anonymous component to the KT in the future.

### How findings compare with existing literature

We used evidence-based methods specific to clinical guideline implementation such as KT lead by a clinician to allow for clinician-to-clinician transfer [29–31], the inclusion of professional decision-support tools [32, 33], presentation of up-to-date systematic reviews rather than only individual research studies [29], and the development of interactive, multi-disciplinary KT interventions as opposed to simply passive interventions [32].

The coding of barriers to guideline implementation that we saw in this project parallel those found in the literature for similar implementation and KT studies [29, 34].

Within the clinical practice field, differences based on context have been noted in success of KT, indicating benefit in designing context-specific KT interventions. In the field of obstetrics, for example, one study concluded that a multifaceted strategy based on audit and feedback that is facilitated by local opinion leaders may be the most effective behaviour change than any single KT intervention [35]. However, even when designed for the appropriate clinical context, KT interventions can be successful in one hospital but not another [36], demonstrating perhaps that further nuances are at play. Indeed, the success of KT is contingent on factors at multiple levels – broad environment, health care system, health care institution, health care team, individual health care provider, and patient – and may run into challenges at any of these levels [37]. As we saw in our analyses, KT success is also influenced by factors outside the individual’s control such as facility and organizational support, and the scope of practice for that individual’s role [38]. Factors related to the learner specifically [38–40], as well as the change being proposed also influence the outcomes of KT interventions. A clear clinical improvement over current behaviour [41] and simplicity versus complexity of the proposed change [37, 41–43] help bring about success. The barriers and facilitators noted by respondents across our three KT formats reflect these complexities.

### Strengths and limitations

The knowledge translation portion of the MAG-CP project has a number of strengths. We used a multi-format approach to the same information, with potential overlap of participants, to allow for representation of different learning styles. Our approach also included clear decision-making tools, which the literature has shown to be helpful in guideline implementation post introduction [17]. Our knowledge translation plan was flexible and allowed for the addition of an examination of facilitators to guideline adoption after the e-learning module had been launched, as well as ongoing revision and redistribution of the B&F survey. We were able to interact with a large number and variety of health care workers, covering coast to coast geographic representation across Canada (4.5 hours of time zones), and reaching what we determined was saturation (for all KT formats) in a relatively short period of time (March 2012 – March 2013).

This project has limitations. First, our data collection does not allow for us to adequately analyze overlap among respondents who may have participated in all three KT strategies, or distinguish who among the B&F survey respondents who indicated ‘inadequate knowledge and understanding’ after occurrence of their site’s visit actually attended that visit. Second, as the e-learning module was presented as aggregate data and the site visit reports were compiled as comprehensive reports (as opposed to verbatim transcripts), we were unable to analyze respondent-specific trends across questions and topics in these formats. Third, a limitation of this study is under-representation of some healthcare provider types whose experience and opinions are relevant. For example, respiratory therapists are not responsible for the decision to administer MgSO4 during labour, but may see effects of interventions on newborns, infants, and children; midwives are not well represented compared with physicians, but may be working with and providing education for women for whom this intervention is relevant. Fourth, the addition of facilitators (as opposed to just barriers) to the data collection was decided after the e-learning module had been launched, preventing comparison of facilitators across all three KT formats. Finally, site visit reports were written as a discussion summary by the Central MAG-CP Team as opposed to direct or aggregate responses from participants seen in the other two formats, which may introduce observer bias, and made comparison across formats less direct.

## Conclusions

With each of the e-learning module, site visits, and local B&F survey, we found advantages and disadvantages such that none was deemed superior for comprehensiveness of respondents reached, barriers and facilitators identified, knowledge felt that they still required, and cost. We would recommend taking all such approaches in future perinatal KT endeavours, although the site visits could be led by local teams to decrease cost.

This information has been given back to sites through presentations and teleconferences with centres. Implementation strategies have been modified to barriers identified, such as creation of new practice tools and reminders. Comparison with data audit is currently ongoing. As audit and feedback are essential parts of the process by which evidence to practice gaps are closed [17], MAG-CP project is continuing the iterative KT process described in this paper in parallel with tracking of MgSO4 use for fetal neuroprotection and maternal and child outcomes until September 2015.

## Availability of supporting data

The data set(s) supporting the results of this article is(are) included within the article (and its additional file(s)).

## Additional files

Additional file 1: Table S1. The MAG-CP (MAGnesium sulphate for prevention of Cerebral Palsy) Collaborative Group. **Table S2:** Ethical approval numbers by site. **Table S3:** Barriers and Facilitators Survey. **Table S4:** Comparison of nodes and sub-nodes between final analysis (by KCT and KC) and re-analysis (by DAD). **Panel S2:** Final NVivo coding list (nodes and sub-nodes) for analysis. **Panel S3:** Nature of knowledge identified as needed by respondents (N (%) responses). (DOCX 101 kb) Additional file 2: Panel S1. Discussion forum and course evaluation questions from the e-learning module for MgSO4 for fetal neuroprotection used in data coding and analysis. (DOCX 13.7 KB)

## Abbreviations

ACOG: American College of Obstetricians and Gynecologists
B&F: barriers and facilitators
BC: British Columbia
BCWH: British Columbia Women’s’ Hospital and Health Centre
CIHR: Canadian Institutes of Health Research
CNFUN: Canadian Neonatal Follow-up Network
COREQ: consolidated criteria for reporting qualitative research
CP: Cerebral Palsy
CPN: Canadian Perinatal Network
HDP: hypertensive disorder of pregnancy
KT: knowledge translation
MAG-CP: magnesium sulphate for fetal neuroprotection of the preterm infant
MD: Medical Doctor
MgSO4: Magnesium Sulphate
RCT: randomized controlled trials
RN: registered nurse
SOGC: Society of Obstetricians & Gynaecologists of Canada
UBC: University of British Columbia
