# Supplementary material for: Magnesium sulphate for fetal neuroprotection: benefits and challenges of a systematic knowledge translation project in Canada
Source: BMC Pregnancy Childbirth. 2015 Dec 22;15:347. doi: 10.1186/s12884-015-0785-8 (PMC4688933; doi:10.1186/s12884-015-0785-8)
Supplement: Additional file 1: Table S1. — The MAG-CP (MAGnesium sulphate for prevention of Cerebral Palsy) Collaborative Group. Table S2: Ethical approval numbers by site. Table S3: Barriers and Facilitators Survey. Table S4: Comparison of nodes and sub-nodes between final analysis (by KCT and KC) and re-analysis (by DAD). Panel S2: Final NVivo coding list (nodes and sub-nodes) for analysis. Panel S3: Nature of knowledge identified as needed by respondents (N (%) responses). (DOCX 101 kb) [file 12884_2015_785_MOESM1_ESM.docx]

**SUPPLEMENTARY APPENDIX**

**Table S1:** The MAG-CP (MAGnesium sulphate for prevention of Cerebral Palsy) Collaborative Group

| **MAG-CP site** | **Location** | **Local team membership** |
| --- | --- | --- |
| **British Columbia Women’s Hospital and Health Centre** | Vancouver, British Columbia | Dane De Silva  Peter von Dadelszen  Laura A. Magee  Anne Synnes |
| **Foothills Medical Centre** | Calgary, Alberta | Stephanie Cooper  Lorel Derderer |
| **Royal Alexandra Hospital** | Edmonton, Alberta | Carmen Young  Cheryl Lux |
| **Royal University Hospital** | Saskatoon, Saskatchewan | Femi Olatunbosun |
| **Regina General Hospital** | Regina, Saskatchewan | George Carson |
| **London Health Sciences Centre** | London, Ontario | Renato Natale  Laura Kennedy |
| **Mount Sinai Hospital** | Toronto, Ontario | Wendy Whittle  Karen Muller |
| **Sunnybrook Health Sciences Centre** | Toronto, Ontario | Noor Ladhani |
| **The Ottawa Hospital** | Ottawa, Ontario | Mark Walker  Melin Peng |
| **Centre Hôspitalier de L’Université Laval** | Quebec City, Quebec | Emmanuel Bujold  Katy Gouin  Amelie Tetu |
| **IWK Health Centre** | Halifax, Nova Scotia | Victoria Allen |
| **Women’s Health Program, Eastern Health** | St. John’s, Newfoundland | Joan Crane  Donna Hutchens  Sandra Adams |
| **Saint John Regional Hospital** | Saint John, New Brunswick | James Andrews |
| **Dr. Everett Chalmers Hospital** | Fredericton, New Brunswick | Kimberly Butt |
| **The Moncton Hospital** | Moncton, New Brunswick | Lynn Murphy-Kaulbeck  Laura Gaudet (previous)  Claire Williams |

**Table S2:** Ethical approval numbers by site

| **MAG-CP site** | **Location** | **Ethics** |
| --- | --- | --- |
| **British Columbia Women’s Hospital and Health Centre** | Vancouver, British Columbia | H11-02214 |
| **Foothills Medical Centre** | Calgary, Alberta | * |
| **Royal Alexandra Hospital** | Edmonton, Alberta | MS4_Pro00030586 |
| **Royal University Hospital** | Saskatoon, Saskatchewan | Bio 12-194 |
| **Regina General Hospital** | Regina, Saskatchewan | REB-13-55 |
| **London Health Sciences Centre** | London, Ontario | 102889 |
| **Mount Sinai Hospital** | Toronto, Ontario | 12-0259-E |
| **Sunnybrook Health Sciences Centre** | Toronto, Ontario | 388-2012 |
| **The Ottawa Hospital** | Ottawa, Ontario | 20130143-01H |
| **Centre Hôspitalier de L’Université Laval** | Quebec City, Quebec | B12-04-943-21 |
| **IWK Health Centre** | Halifax, Nova Scotia | 1004849 |
| **Women’s Health Program, Eastern Health** | St. John’s, Newfoundland | 12.100 |
| **Saint John Regional Hospital** | Saint John, New Brunswick | 2013-1816 |
| **Dr. Everett Chalmers Hospital** | Fredericton, New Brunswick | 2012-1735 |
| **The Moncton Hospital** | Moncton, New Brunswick | 2012-1727 |

***** The one site that participated only in the educational study visit (but not data collection) decided not to proceed with the rest of the study and they did not pursue local REB approval.

**Table S3:** Barriers and Facilitators Survey

| **Assessment of barriers and facilitators to practice change** |
| --- |
| As part of the MAG-CP implementation project, it is important for us to identify and assess factors that may either help to change practice (a ‘facilitator’) or hinder changes in practice (a ‘barrier’) in your centre. These factors may be characteristics of: individual care providers, relationships between care providers, or the organizational culture of your hospital. |
| Thank you in advance for your anonymous input! |
| **1. Do you feel that you have adequate KNOWLEDGE and UNDERSTANDING of the EVIDENCE for magnesium sulphate for fetal neuroprotection?** |
| Yes  No 🡪 If no, please tell us what you would like to know more about: _______________ |
| ______________________________________________________________________________ |
| **2. Do you believe that the evidence is rigorous enough to warrant use of magnesium sulphate for fetal neuroprotection in clinical practice?** |
| No  Yes  Don’t know 🡪 If no, please share with us your concerns: __________________ |
| ______________________________________________________________________________ |
| **3. Are you aware of an opinion leader in your centre who has strong views about use of magnesium sulphate for fetal neuroprotection?** |
| No  Yes 🡪 if yes, does he/she believe that magnesium sulphate should be used for fetal neuroprotection?  Yes  No |
| **4. Do you consider yourself to be an ‘early adopter’ of evidence?** |
| No  Yes  Don’t know |
| **5. Do you consider yourself to be a ‘late adopter’ of evidence?** |
| No  Yes  Don’t know |
| **6. Do you feel that you have adequate KNOWLEDGE and UNDERSTANDING of the 2011 SOGC Guideline on use of magnesium sulphate for fetal neuroprotection?** |
| No  Yes |
| **7. Are you concerned about medico-legal problems related to use magnesium sulphate for fetal neuroprotection?** |
| No  Yes 🡪 if yes, please describe for us your concerns: _____________________________ |
| ______________________________________________________________________________ |
| **8. Are you aware of women in your centre who have asked for magnesium sulphate for fetal neuroprotection?**  No  Yes |
| **9. Are you aware of an institutional policy at your centre for use of magnesium sulphate for fetal neuroprotection?** |
| No  Yes 🡪 if yes, does this policy recommend use of magnesium sulphate for fetal neuroprotection in the setting of preterm birth at <32 weeks?  No  Yes  Don’t know |
| **10. Do you have pre-printed orders for use of magnesium sulphate for fetal neuroprotection?**  No  Yes |
| **11. For use of magnesium sulphate for fetal neuroprotection in the setting of ‘imminent preterm birth’ at <32 weeks, how concerned are you about the resource implications in the following areas of the hospital:** |
| **Delivery suite** |
| Not at all concerned  Somewhat concerned  Concerned  Very concerned  Extremely concerned |
| **Neonatal intensive care unit** (NICU) |
| Not at all concerned  Somewhat concerned  Concerned  Very concerned  Extremely concerned |
| **Pharmacy** |
| Not at all concerned  Somewhat concerned  Concerned  Very concerned  Extremely concerned |
| **12. Do you feel that in your centre there is administrative support for use of magnesium sulphate for fetal neuroprotection?**  Yes  No  Don’t know |
| **13. Please share with us any characteristics of your organization that you feel may HELP to implement the SOGC clinical practice guidelines on magnesium sulphate for fetal neuroprotection.** _______________________________________________________________ ______________________________________________________________________________ |
| **14. Please share with us any characteristics of your organization that you feel may HINDER the implementation of the SOGC clinical practice guidelines on magnesium sulphate for fetal neuroprotection.** ___________________________________________________________ ______________________________________________________________________________ |
| **15. Please share with us any other comments that you feel would be helpful.** _____________ |
| ______________________________________________________________________________ |
| Thank you in advance for answering two brief questions about yourself. |
| **16. What clinical group do you represent?** *Please choose the ONE best answer.* |
| MFM  General obstetrics and gynaecology  Neonatology  Anaesthesia  Pharmacy |
| Midwifery  General practice  Nursing  Other (please specify) ___________________ |
| **17. What do you spend at least 50% of your time doing?** *Please check the ONE best answer.* |
| Clinical work (≥50%)  Research (≥50%)  Administration (≥50%)  Education (≥50%) |
| Other (please specify)__________________________________________________________ |

**Table S4:** Comparison of nodes and sub-nodes between final analysis (by KCT and KC) and re-analysis (by DAD)

|  | **Nodes** | **Sub-nodes** | **Corresponding node (KC/KCT)** |
| --- | --- | --- | --- |
| **Barriers** | | | |
| **Individual-level** | | | |
|  | Attitudes and beliefs |  | Unsupportive attitudes and beliefs |
|  | Practices & Habits |  | Forgetting to administer MgSO4 |
|  | Inadequate knowledge and understanding |  | Inadequate knowledge and understanding |
|  |  |  |  |
|  |  |  |  |
|  | Fears & Medico-legal |  | Fears |
| **Institutional-level** | | | |
|  | Organizational culture |  | Unsupportive institutional culture |
|  | Resource constraints |  | Resource constraints |
| **Facilitators** | | | |
| **Individual-level** | | |  |
|  | Attitudes and beliefs |  | Supportive attitudes and beliefs |
| **Institutional-level** | | | |
|  | Administrative support |  | Local champion/opinion leader |
|  |  |  |  |
|  |  |  |  |
|  | Organizational culture | Collaboration | Facility characteristics |
|  |  | Institutional policy | Policies and protocols |
| **Social-level** | | | |
|  | Educational activities |  | Knowledge translation |
| **Knowledge Needed** | | | |
|  | Evidence & education | Further research | Research |
|  |  | Evidence to-date |  |
|  | Mechanism of action |  | Mechanism of action |
|  | Practices & Habits |  | Administration |

**Panel S2:** Final NVivo coding list (nodes and sub-nodes) for analysis

| **Nodes** | | **Sub-nodes** |
| --- | --- | --- |
| **BARRIERS** | | |
| **Individual-level** | | |
|  | Unsupportive attitudes and beliefs | |
|  | Not within provider’s control | |
|  | No experience | |
|  | Inadequate knowledge and understanding* | Inadequate knowledge/understanding in self |
|  |  | Inadequate knowledge/understanding in others |
|  |  | Unclear who respondent is referring to |
|  | Forgetting to administer MgSO_4_ | |
|  | Fears | Legal |
|  |  | Medication error |
|  |  | Adverse effects of withholding MgSO4 |
|  |  | Adverse effects of MgSO4 |
|  | Failure to implement guidelines | |
|  | Evidence concerns (sufficiency and validity) | |
| **Institutional-level** | | |
|  | Unsupportive institutional culture | |
|  | Timing and transport | |
|  | Resource constraints | |
|  | Policy development and implementation | |
| **Social-level** | | |
|  | Lack of provider-institutional consensus | |
|  | Inadequate inter-provider communication | |
|  | Educating patients | |
| **FACILITATORS** | | |
|  | **Individual-level** | |
|  | Supportive attitudes and beliefs | |
|  | Knowledge and understanding | |
|  | Early adopters/mobilizers | |
|  | Comfort/experience using MgSO_4_ | |
|  | **Institutional-level** | |
|  | Policies and protocols | Pre-printed orders for MgSO4 use |
|  |  | Pre-mixed bags of MgSO4 |
|  |  | Mechanism for audit and feedback |
|  | Local champion/opinion leader | |
|  | Facility characteristics | Supportive institutional culture/evidence-based |
|  |  | Patient load |
|  |  | Human resource capacity |
|  |  | Education and professional development |
|  | **Social-level** | |
|  | Patient voice/awareness | |
|  | Knowledge translation | |
|  | Community support | |
|  | Communication and collaboration | |

** Please see Panel 3 for details about the nature of knowledge needed.*

**Panel S3:** Nature of knowledge identified as needed by respondents (N (%) responses)

| **Nodes** | | **Sub-nodes** | **Sub-sub-node** |
| --- | --- | --- | --- |
|  | **Mechanism of action** | | |
|  | **Administration** | Transfer | |
|  |  | Threatened preterm labour vs. imminent preterm birth | |
|  |  | Timing of administration | |
|  |  | Standards of practice | |
|  |  | Re-treatment | |
|  |  | Pre-printed orders | |
|  |  | Policies and protocols | |
|  |  | Multiple pregnancies | |
|  |  | Gestational age | |
|  |  | Drug interactions | |
|  |  | Contraindications | |
|  | **Side effects and risks** | Rapid delivery | |
|  |  | Overuse | |
|  |  | Interventions as a result of MgSO4 | |
|  |  | Increased monitoring needed | |
|  |  | Adverse physiological effects – neonate | Toxicity |
|  |  |  | Problems with feeding |
|  |  |  | Neonatal respiratory depression |
|  |  |  | Long-term effects |
|  |  |  | Adverse neurological effects |
|  |  | Adverse physiological effects – general (unspecified maternal/neonate) | |
|  | **KT tools** | Audit and feedback | |
|  | **Research** | Further research | |
|  |  | Evidence to date | |
|  | **Other uses and topics (not MgSO_4_ for fetal neuroprotection)** | | |
|  | **None stated** | | |

*MgSO_4_ (magnesium sulphate)*
