# Supplementary material for: Magnesium sulphate for fetal neuroprotection: benefits and challenges of a systematic knowledge translation project in Canada
Source: BMC Pregnancy Childbirth. 2015 Dec 22;15:347. doi: 10.1186/s12884-015-0785-8 (PMC4688933; doi:10.1186/s12884-015-0785-8)
Supplement: Additional file 2: Panel S1. — Discussion forum and course evaluation questions from the e-learning module for MgSO4 for fetal neuroprotection used in data coding and analysis. (DOCX 13.7 KB) [file 12884_2015_785_MOESM2_ESM.docx]

**Appendix**

**Panel S1:** Discussion forum and course evaluation questions from the e-learning module for MgSO4 for fetal neuroprotection used in data coding and analysis

| **Discussion forum questions** |
| --- |
| Is there any additional information that you would like to have before using magnesium sulphate for fetal neuroprotection in your practice? If ‘yes, please explain. |
| Do you perceive any barriers that may prevent use of magnesium sulphate for fetal neuroprotection? If ‘yes’, please explain. |
| Are you concerned about the effects of magnesium sulphate for fetal neuroprotection on the baby in the newborn period? If ‘yes, please explain. |
| Following completion of this programme, are you likely to use magnesium sulphate for fetal neuroprotection? |
| **Course evaluation questions** |
| Can you identify changes you would make in your practice as a result of this program? |
| Are there any areas related to this topic that you’d like to learn more about in future programs? |

*MgSO4 (magnesium sulphate)*
